# Cardiomyocyte-specific role of miR-24 in promoting cell survival

**DOI:** 10.1111/jcmm.12393

**Published:** 2014-10-29

**Authors:** Chuner Guo, Yangmei Deng, Jiandong Liu, Li Qian

**Affiliations:** a Department of Pathology and Laboratory Medicine, University of North CarolinaChapel Hill, NC, USA; b McAllister Heart Institute, University of North CarolinaChapel Hill, NC, USA; c Lineberger Comprehensive Cancer Center, University of North CarolinaChapel Hill, NC, USA

**Keywords:** cardiomyocyte, microRNA, apoptosis, myocardial infarction

## Abstract

Cardiomyocyte cell death is a major contributing factor to various cardiovascular diseases and is therefore an important target for the design of therapeutic strategies. More recently, stem cell therapies, such as transplantation of embryonic or induced pluripotent stem (iPS) cell-derived cardiomyocytes, have emerged as a promising alternative therapeutic avenue to treating cardiovascular diseases. Nevertheless, survival of these introduced cells is a serious issue that must be solved before clinical application. We and others have identified a small non-coding RNA, microRNA-24 (miR-24), as a pro-survival molecule that inhibits the apoptosis of cardiomyocytes. However, these earlier studies delivered mimics or inhibitors of miR-24 *via* viral transduction or chemical transfection, where the observed protective role of miR-24 in cardiomyocytes might have partially resulted from its effect on non-cardiomyocyte cells. To elucidate the cardiomyocyte-specific effects of miR-24 when overexpressed, we developed a genetic model by generating a transgenic mouse line, where miR-24 expression is driven by the cardiac-specific *Myh6* promoter. The Myh6-miR-24 transgenic mice did not exhibit apparent difference from their wild-type littermates under normal physiological conditions. However, when the mice were subject to myocardial infarction (MI), the transgenic mice exhibited decreased cardiomyocyte apoptosis, improved cardiac function and reduced scar size post-MI compared to their wild-type littermates. Interestingly, the protective effects observed in our transgenic mice were smaller than those from earlier reported approaches as well as our parallelly performed non-genetic approach, raising the possibility that non-genetic approaches of introducing miR-24 might have been mediated *via* other cell types than cardiomyocytes, leading to a more dramatic phenotype. In conclusion, our study for the first time directly tests the cardiomyocyte-specific role of miR-24 in the adult heart, and may provide insight to strategy design when considering miRNA-based therapies for cardiovascular diseases.

## Introduction

One of the immediate consequences of cardiac injury is cell death. In a single event of myocardial infarction (MI), more than 25% of cardiomyocytes can be wiped out [1]. The massive death of the contractile muscle cells is followed by scar formation, dysfunction of the heart and ultimately heart failure. Because the human heart has a limited regenerative capacity, preventing the loss of existing cells and promoting the generation of new cardiomyocytes are current approaches being extensively studied.

Recent advances in direct cardiac reprogramming introduce a new possibility of regenerating the heart by converting endogenous cardiac fibroblasts (CFs) or other fibroblasts into cardiomyocyte-like cells (mouse, [2–6]; human, [7–9]). The potential to utilize a large pool of endogenous CFs to generate functional cardiomyocytes points to a promising avenue to repairing the damaged myocardium in human patients. However, the fact remains that most of the reprogrammed CFs do not become fully matured, and that *in vivo* reprogramming has only worked thus far when the reprogramming factors (such as Gata4, Mef2c and Tbx5 (GMT) in mice) are introduced at the time of injury.

Another approach to regenerating the heart is to derive new cardiomyocytes for transplantation. These cells are typically differentiated from multipotent cardiovascular progenitor cells (CPCs) [10,11] or pluripotent stem cells, including both embryonic stem (ES) cells [12–15] and induced pluripotent stem (iPS) cells [16,17]. Although efforts to make this a practical therapy are ongoing, the poor survival, low maturational efficiency, limited cell–cell interaction and poor functional integration of engrafted cells are major hurdles that remain to be overcome [1].

Alternatively, researchers are actively searching for factors that regulate cardiomyocyte proliferation [18–20] and cell death [21–24] to facilitate the regeneration of injured hearts. microRNAs (miRNAs) are small non-coding RNAs that are typically 20-22 nucleotides in length and play important roles in many biological processes [25–27]. Because miRNAs' short lengths facilitate easier and safer delivery, they are suitable candidates and/or targets for disease therapies. However, the regulatory network of one microRNA can be rather complicated, typically involving multiple targets by binding to their 3′ UTR regions *via* its ‘seed region’. A complete understanding of one microRNA's functions under different contexts is, therefore, extremely important for proper design of therapeutic strategies to avoid potential side effects.

The function of miR-24 in the heart is a good example of the potential complexity of a single miRNA's regulatory role. On the one hand, miR-24 has been reported to be pro-survival. It has been shown that miR-24 inhibits apoptosis in cardiomyocytes, and that introduction of miR-24 into the heart significantly attenuates cardiac dysfunction [24]. miR-24 was also found to promote CPC's functional engraftment in a study led by Hu *et al*., where miR-24 was used as part of an anti-apoptotic cocktail to promote the survival of stem cell-derived CPCs for transplantation into MI hearts [28]. Additional pro-survival roles of miR-24 in cardiac fibrosis were identified by Wang and colleagues, who found that overexpression of miR-24 in the MI heart by lentivirus-mediated transduction reduced fibrosis and improved heart function, confirming the beneficial role of miR-24 when expressed under acute injury [29]. On the other hand, pro-apoptotic roles of miR-24 were also reported. miR-24 was characterized as a pro-apoptotic microRNA in endothelial cells in the heart, where injection of antagomir of miR-24 that blocks its function prevented endothelial apoptosis, enhanced vascularity and resulted in an improved heart function after MI [30]. In a mouse hypertrophy model where the aorta was constricted artificially, suppression of miR-24 protected the heart from transitioning from compensated hypertrophy to decompensated hypertrophy through regulation of L-type Ca^2+^ channel-ryanodine receptor signalling [31]. Therefore, the context-dependent function of miR-24 in apoptosis is intriguing and worthy of further investigation. Importantly, although significant advances have been made in understanding the role of miR-24, there had been no simple genetic model to precisely and directly test the cardiomyocyte-specific role of miR-24 in the heart.

In this article, we generated a cardiac-specific transgenic mouse model of miR-24, in which we utilized the *Myh6* promoter to drive the expression of miR-24 in cardiomyocytes in the heart. We report here that *Myh6* promoter-driven miR-24 expression was sufficient to promote cardiomyocyte survival post-acute injuries. In addition, cardiac overexpression of miR-24 resulted in scar size reduction and heart function improvement. Furthermore, we investigated the molecular pathway through which miR-24 functions and showed that miR-24 modulates intrinsic apoptosis in our transgenic model. Although a milder effect was observed compared to the results from a non-genetic approach [24], our findings support the anti-apoptotic role of miR-24 in the heart, and suggest that additional effects may be mediated through cell types other than cardiomyocytes.

## Materials and methods

### Mouse lines

To generate the Myh6-miR-24 mice, a cDNA construct containing a 400-bp mouse genomic region encompassing the miR-24-2 locus was cloned downstream of the mouse cardiac myosin heavy chain (α*MHC*/*Myh6*) promoter. Primers used for cloning were: forward primer 5′-CACCATCTCCTCAGGCCGCTGCTG-3′, reverse primer 5′-CTATCTGCTTTGGGGAACCACAG-3′. Transgenic mice were produced by microinjection of the Myh6-miR-24 construct into fertilized mouse embryos (C57BL/6 background). Three independent transgenic lines were established and one line with a sixfold overexpression of miR-24 was extensively studied. Transgenic mice were identified by PCR analysis of tail genomic DNA using the forward primer 5′-GCCCACACCAGAAATGACAGA-3′ and the reverse primer 5′-CTATCTGCTTTGGGGAACCACAG-3′. Functional studies, gene expression and immunohistochemistry (IHC) were analysed in pairs of Myh6-miR-24 (TG) and littermate non-transgenic (WT) male mice.

### Immunohistochemistry

After perfusion fix with 4% paraformaldehyde (PFA, diluted from 20% PFA stock, Electron Microscopy Sciences) in PBS, the heart was removed and fixed by immersion in 4% PFA in PBS overnight at 4°C and routinely processed and paraffin embedded. Sections (5 μm) were stained with haematoxylin and eosin and analysed for regular morphology. To measure the infarct size at 4 weeks after MI, left ventricles (LVs) were cut from apex to base; sections from four equally distributed levels were AZAN- or Masson-Trichrome stained. Scar size was calculated as the percentage of the LV circumference, or total scar area divided by total LV area, and was summed from four transverse sections per heart. To quantify apoptotic cardiomyocytes, mouse hearts were removed 24 hrs after coronary artery ligation and were fixed with 0.5% PFA in 5% sucrose and routinely frozen embedded in O.C.T and processed for sectioning and staining with Caspase3 (rabbit, 1:200; Sigma-Aldrich, St. Louis, MO, USA) or TUNEL and α-Actinin (mouse, 1:800; Sigma-Aldrich) as described [24]. TUNEL was performed with an In Situ Cell Death Detection Kit, Fluorescein (Roche, Nutley, NJ, USA), per the manufacturer's protocol. DAPI was used for nuclear counterstaining. Frozen heart sections were also stained with phospho-histone H3 (rabbit, 1:100; Millipore-Upstate, Billerica, MA, USA), α-Actinin, and DAPI to quantify proliferating cardiomyocytes; alternatively, sections were stained with Wheat Germ Agglutinin (WGA; Alexa Fluor® 488 Conjugate, Life Technology, Carlsbad, CA, USA, per the manufacturer's protocols), α-Actinin, and DAPI to quantify cardiomyocyte size.

### *In vivo* delivery of miR-24 mimic

With the chest open, oligos stabilized with 2′-O-methyl modification pre-treated with 20 μl of lipofectamine 2000 (Invitrogen, Carlsbad, CA, USA) were injected into the myocardium through an insulin syringe with incorporated 29G needle (BD Biosciences, San Jose, CA, USA) into the myocardium. One injection with a full dosage (40 ng of miR-24 mimic or 40 ng of control mimic, ordered from Shanghai GenePharma Co., Shanghai, China) was employed along the boundary between the IZ and BZ (based on the blanched infarct area) after coronary artery occlusion. After injection, the chest was closed with sutures and the mouse was allowed to recover with mouse ventilator and heating pad.

### Echocardiography

Echocardiography was performed with the Vevo 770 High-Resolution Micro-Imaging System (VisualSonics, Toronto, ON, Canada) with a 15-MHz linear array ultrasound transducer. The LV was assessed in both parasternal long-axis and short-axis views at a frame rate of 120 Hz. End-systole or end-diastole was defined as the phase in which the smallest or largest area of LV, respectively, was obtained. Left ventricular end-systolic diameter (LVDS) and left ventricular end-diastolic diameter (LVDD) were measured from the LV M-mode tracing with a sweep speed of 50 mm/sec. at the papillary muscle level.

### LAD ligation

All surgeries and subsequent analyses were performed in a blinded fashion for genotype and intervention. Mice were anaesthetized with 2.4% isoflurane/97.6% oxygen and placed in a supine position on a heating pad (37°C). Animals were intubated with a 19G stump needle and ventilated with room air with a MiniVent Type 845 mouse ventilator (Hugo Sachs Elektronik-Harvard Apparatus, March-Hugstetten, Germany; stroke volume (SV) 250 μl, respiratory rate 120 breaths/min.). MI was induced by permanent ligation of the left anterior descending artery (LAD) with a 7-0 prolene suture as described [32]. Sham-operated animals served as surgical controls and were subjected to the same procedures as the experimental animals with the exception that the LAD was not ligated. All surgical procedures were performed under aseptic conditions. Four weeks or 24 hrs after occlusion, the heart was removed for perfusion fix (4% PFA; paraffin sections for structural analysis and IHC) or immersion fix (0.5% PFA in 5% sucrose; cryo sections for immunofluorescent staining); alternatively, the tissues within the infarct zone (IZ), border zone (BZ) and non-ischaemic zone distal to the infarct zone (DZ) were dissected for RNA or protein isolation.

### Area at risk and infarct size

Twenty-four hours after coronary ligation, the mice were anaesthetized and cannulated with tubing. A 2% Evans Blue solution (Sigma-Aldrich) was perfused into the aorta, thus all myocardial tissue except the AAR was stained blue. The LV was isolated and cut into four ∼1 mm pieces with first cut at the ligation level. LV slices were stained in 1.5% triphenyltetrazolium chloride (TTC) for 30 min. at 37°C, and then fixed in 4% PFA overnight at 4°C. The area of infarction was demarcated as a white area, while viable myocardium was stained red. Photographs were taken for both sides of each section. The AAR and the infarct area were determined *via* planimetry by using computer software ImagePro (Bio-Rad, Hercules, CA, USA). Infarct size was calculated as the percentage of MI compared with the AAR using a previously described methodology [33].

### Real-time PCR

RNA was extracted using TRizol (Invitrogen). RT-PCR was performed with the Superscript III first-strand synthesis system (Invitrogen). qPCR was performed with the ABI 7900HT (TaqMan, Applied Biosystems, Carlsbad, CA, USA), per the manufacturer's protocols. Optimized primers from Taqman Gene Expression Array were used. miRNA RT was conducted using the Taqman MicroRNA Reverse Transcription Kit (Applied Biosystems). miRNA real-time PCR (qRT-PCR) was performed per the manufacturer's protocols by using primers from Taqman MicroRNA Assays (Applied Biosystems). Expression levels were normalized to GAPDH expression and miR-16 (microRNA qPCR) as previously reported [24].

### Western blot

Western blots were performed as described [34]. Briefly, proteins were separated by gel electrophoresis, transferred to the Hybond-P PVDF membrane, blocked with 2% milk in PBST, incubated with primary and HRP secondary antibodies, treated with luminol substrate and developed on a film. Mouse monoclonal anti-caspase 8 (Sigma-Aldrich), mouse monoclonal anti-caspase 9 (Sigma-Aldrich), rabbit anti-caspase 3 (Sigma-Aldrich), rat monoclonal anti-caspase 12 (Sigma-Aldrich) and rabbit polyclonal antibody against Bim (amino acids 4–195 of Bim_EL_ form) were all used at a 1:1000 dilution for Western blots.

### Statistics

For echocardiography, TUNEL, Azan, Masson-Trichrome, Evans blue/TTC staining quantification, statistical analysis was performed with SPSS v.15. Comparisons between groups were made by one-way anova or Mann–Whitney *U*-test, as applicable. Sample numbers were indicated in corresponding figures. For qPCR, we used biological triplicates and technical triplicates; data were analysed by unpaired Student's *t*-test. Error bars indicate standard error of the mean (SEM). *P < 0.05, **P < 0.01.

## Results

### Generation of Myh6-miR-24 transgenic mice

**Fig. 1:**
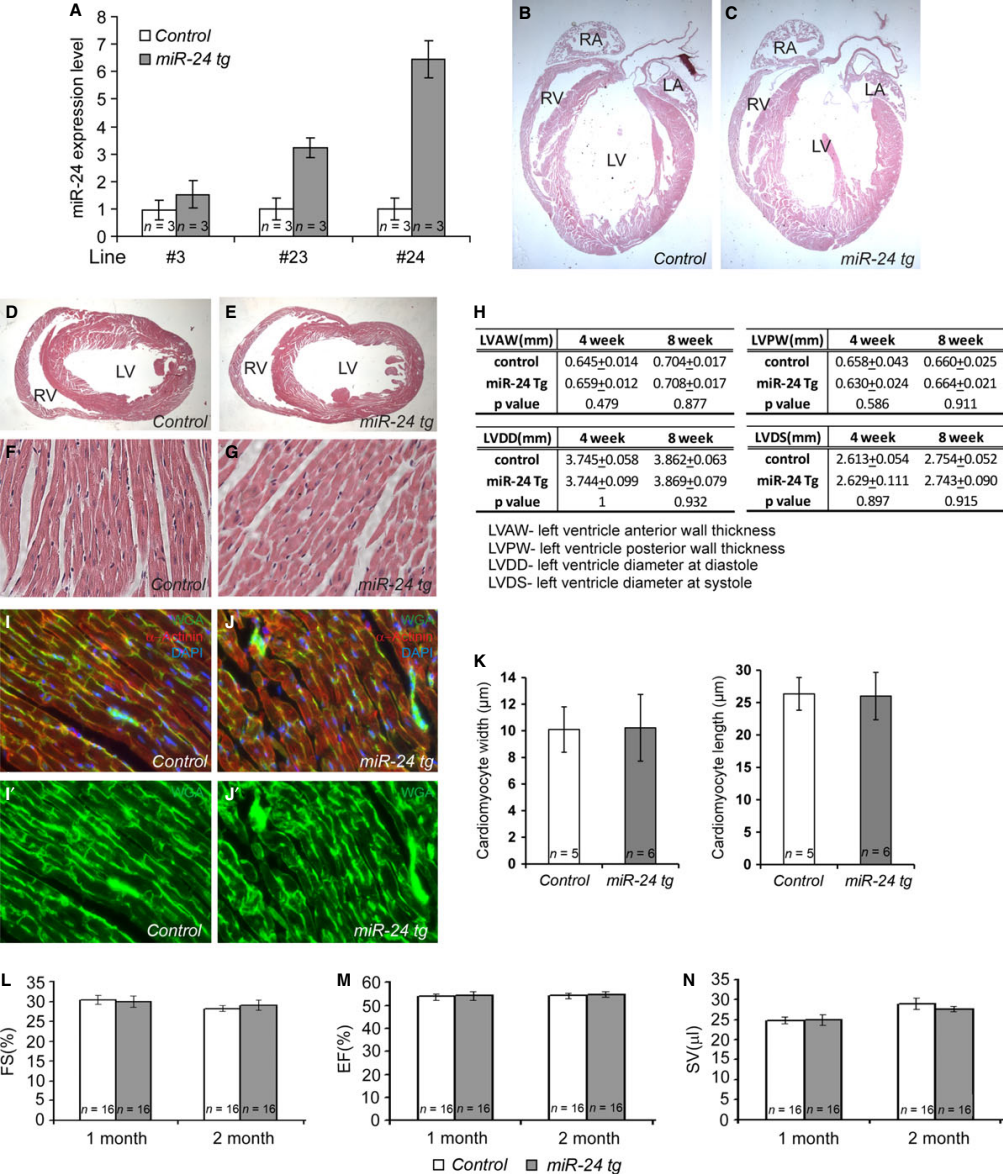
Myh6-miR-24 transgenic mice exhibit elevated miR-24 expression, with no morphological or functional alteration under normal physiological conditions. (**A**) Histogram showing miR-24 overexpression in three independent transgenic (tg) lines. Line #24 with sixfold overexpression was chosen for extensive studies. *n* = 3 for each genotype. (**B**–**E**) Four-chamber and transverse sections with haematoxylin and eosin staining on control and miR-24 hearts, both showing normal morphologies. (**F** and **G**) High magnification of sections from control (*n* = 5) and miR-24 tg (*n* = 6) hearts, both showing normal cell morphology. (**H**) Table showing normal heart shape in both control (*n* = 16) and miR-24 tg (*n* = 16) mice. (**I**–**J'**) Immunohistochemistry with WGA (green), α-Actinin (red) and DAPI counterstain (blue) on control and miR-24 tg heart sections. (**K**) Quantification of cardiomyocyte width and length of control (*n* = 5) and miR-24 tg (*n* = 6) heart, showing no significant difference. (**L**–**N**) Histograms showing normal cardiac function in both control (*n* = 16) and miR-24 tg (*n* = 16) mice. FS, fractional shortening; EF, ejection fraction; SV, stroke volume.

Previous studies revealed critical roles of miR-24 in promoting cell survival, especially in murine cardiomyocytes, suggesting a potential therapeutic application [24]. However, all of the *in vivo* studies so far used non-genetic approaches, such as viruses and synthetic oligonucleotides, to overexpress or inhibit miR-24. These approaches could potentially affect other cell types including fibroblasts, endothelial cells, circulating inflammatory cells and haematopoietic cells. To investigate whether miR-24 exerts its effects primarily in the cardiomyocytes to promote cell survival, we genetically targeted miR-24 under control of the cardiac-specific *Myh6* promoter, which is predominantly activated after embryonic cardiac development [35]. Expression of mature miR-24 was quantified by qPCR, which revealed that the transgenic mice had significantly higher levels of miR-24 expression than their control littermates (Fig. 1A). For detailed analysis, we chose the line with a sixfold greater expression of miR-24, an overexpression level not too high to overload the microRNA machinery within the heart.

### miR-24 transgenic mice exhibited no significant difference from their littermates under normal physiological conditions

We first determined if a moderate overexpression of miR-24 in adult cardiomyocytes could result in any morphological alteration in the heart. Specifically, we examined the transgenic and wild-type control hearts from mice ranging in age from 1 day to 10 weeks. Haematoxylin and eosin staining on both 4-chamber view sections and transverse sections revealed normal cardiac structures in the Myh6-miR-24 transgenic mice compared to the littermate controls (Fig. 1B and C). In addition, we quantified the length of LV anterior wall and LV posterior wall to determine if overexpression of miR-24 in cardiomyocytes could lead to wall thickening or thinning, and did not identify such events (Fig. 1H, top).

When examined at high magnifications, cells within the hearts of the transgenic mice exhibited normal morphologies (Fig. 1F and G). To determine if there is difference in cardiomyocyte size and gross sarcomere assembly, we co-stained heart sections with WGA, α-Actinin and DAPI, and performed blinded quantification using stained sections from four levels of LV. As shown in Figure 1I–K, no significant difference was detected in cardiomyocyte size between transgenic and littermate control mice, neither was the gross sarcomere assembly significantly different. These mice did not develop hypertrophy, hyperplasia, or heart failure when aged to 1–2 years (before they died).

Next, we asked if expression of miR-24 in cardiomyocytes would lead to any changes in heart function. We performed high-resolution two-dimensional echocardiography on transgenic and control mice when they were 1 and 2 months old (Fig. 1L–N). The fractional shortening (FS) of the ventricular chamber (FS) was not altered in transgenic mice compared to wild-type littermates (Fig. 1L); neither was the fraction of blood ejected from the LV with each contraction (ejection fraction, EF) altered (Fig. 1M). Using an additional parameter to assay the functional aspects of the heart, we determined the volume of blood ejected with each heart beat (SV), and found no difference between miR-24 transgenic mice and wild-type controls (Fig. 1N). Furthermore, we measured the diameter of the left ventricle during diastole (LVDD) and the diameter of the left ventricle during systole (LVDS) on transgenic and control mice to further support our functional measurements of EF, FS and SV. None of these parameters showed significant difference between transgenic and wild-type mice (Fig. 1H, bottom). These data indicate that cardiac miR-24 overexpression did not interfere with normal cardiac function and structure under normal physiological conditions.

### miR-24 promotes the survival of cardiomyocytes cell autonomously upon injury

**Fig. 2:**
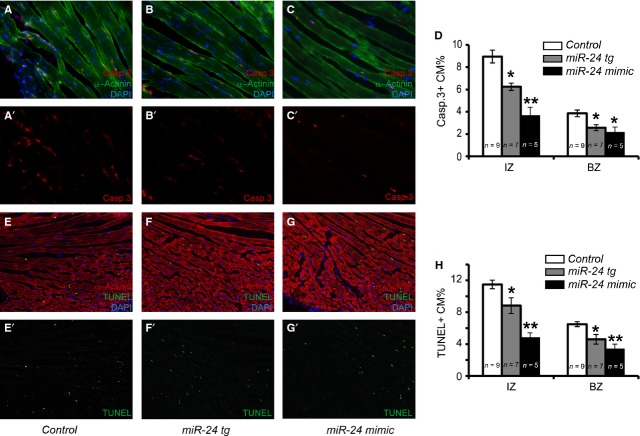
Myh6-miR-24 transgenic mice exhibit reduced cardiomyocyte apoptosis upon myocardial infarction. (**A**–**C'**) Immunocytochemistry with Caspase 3 (red) and α-Actinin (green) labelling as well as DAPI counterstain (blue) on control (*n* = 9), miR-24 tg (*n* = 7) and miR-24 mimic-treated (*n* = 5) hearts. (**D**) Quantification of apoptotic cardiomyocytes (Caspase 3- and α-Actinin-positive) in the infarct zone (IZ) and border zone (BZ), with miR-24 tg and mimic-treated hearts showing reduced apoptosis. (**E**–**H**) Alternative staining and quantification of apoptotic cardiomyocytes (TUNEL- and α-Actinin-positive) in IZ and BZ, with miR-24 tg and mimic-treated hearts showing reduced apoptosis. *P < 0.05. **P < 0.01.

Our previous study using viral delivery of miR-24 mimics has shown that miR-24 inhibits cardiomyocytes apoptosis both *in vitro* and *in vivo* [24]. To follow-up, we wanted to determine if the anti-apoptotic role of miR-24 is exerted directly on cardiomyocytes or through other cell types. We combined Caspase 3 labelling with the cardiomyocyte marker α-Actinin on infarcted mouse hearts from Myh6-miR-24 transgenic, wild-type littermate controls and mice treated with miR-24 mimics (Fig. 2A–C'). The percentages of Caspase 3-positive (apoptotic) cardiomyocytes in the IZ and BZ were lower in transgenic animals than in control littermate mice, but with a milder effect compared to miR-24 mimics (Fig. 2D). This result was further confirmed by using another apoptotic marker, TUNEL, in combination with α-Actinin (Fig. 2E–G' and H). We detected no mitotic cardiomyocytes in either transgenic or littermate control mice (data not shown). Compared to miR-24 expression using synthetic mimics, the anti-apoptotic effects were milder in our transgenic mouse model (50–60% reduction *versus* 20–30% reduction in % of apoptotic cardiomyocytes), suggesting that non-autonomous function of miR-24 when delivered into other cells (*e.g*. CFs) may help promote the survival of cardiomyocytes upon injury. In fact, we have shown in the past that miR-24 is also expressed in CFs but not in endothelial cells [24]. *In situ* hybridization using miR-24 LNA probe and qPCR further demonstrated that expression of miR-24 in myocardium was sharply reduced upon MI, but restored with miR-24 mimic injection [24]. To further compare how our genetic model differs from chemical delivery of miR-24 upon MI, we measured miR-24 levels at injected area, border area and distant area in both Myh6-miR-24 transgenic and miR-24 mimic-injected hearts (Fig. S1). In the transgenic hearts, miR-24 had an even overexpression level, while in mimic-injected hearts, miR-24 had a concentrated overexpression in the injected area but an almost undetectable overexpression in the distant area (Fig. S1). miR-24 mimic injection resulted in a (locally) higher overexpression compared to the transgenic model, which might explain the stronger rescue in myocyte death. Nevertheless, our data support the notion that miR-24 exerts at least part of its *in vivo* anti-apoptotic function directly on cardiomyocytes.

### Improved heart function in infarcted Myh6-miR-24 transgenic mice

Myocardial infarction usually leads to functional decompensation and heart failure after LV remodelling and cardiac fibrosis. We reasoned that if miR-24 prevents the cardiomyocytes from dying, its overexpression in cardiomyocytes themselves would translate into preservation of heart function. Therefore, we performed high-resolution two-dimensional echocardiography to compare the changes in cardiac function after MI between transgenic and wild-type control mice.

**Fig. 3:**
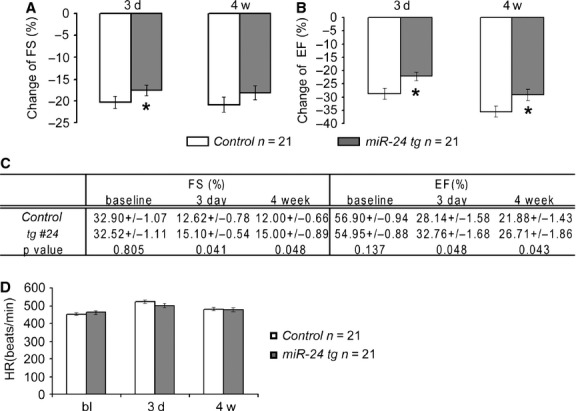
Myh6-miR-24 transgenic mice exhibit improved heart function upon myocardial infarction. (**A** and **B**) Histograms showing reduction in cardiac function in control (*n* = 21) and miR-24 tg (*n* = 21) hearts 3 days and 4 weeks after myocardial infarction, with miR-24 tg hearts showing alleviated reduction in cardiac function. (**C**) Baseline and post-MI (3 days and 4 weeks) measurements of FS and EF in control (*n* = 21) and miR-24 tg (*n* = 21) mice. miR-24 tg mice exhibit better heart function compared to controls. (**D**) Baseline and post-MI (3 days and 4 weeks) measurements of heart rate (HR) in control (*n* = 21) and miR-24 tg (*n* = 21) mice, showing no significant difference between the two. *P < 0.05.

All mice showed initial reduction in LV function (FS and EF) after coronary artery ligation, confirming successful induction of MI, while the miR-24 transgenic mice showed alleviated reduction in all parameters (Fig. 3A and B, Fig. S2A and B). Three days post-MI, miR-24 transgenic mice showed a higher FS (15.10 + 0.54% *versus* 12.62 + 0.78%, *P* < 0.05) and EF (32.76 + 1.68% *versus* 28.14 + 1.58%, *P* < 0.05) compared to control littermates (Fig. 3C). Moreover, SV was significantly higher in transgenic mice than controls (Fig. S2C). As an important control, we measured heart rate (HR) and found no difference between transgenic and control mice (Fig. 3D), suggesting that the improvement in heart function is because of better structure and contractility of the LV.

To test if functional preservation persists over time, we performed the same measurements at 4 weeks post-MI (Fig. 3A–C, Fig. S2A–C). The absolute numbers of FS and EF remained statistically different between transgenic and controls (Fig. 3C, Fig. S2A and B). Furthermore, the difference in SV became more significant between two groups (Fig. S2C), suggesting that functional protection by overexpression of miR-24 in cardiomyocytes lasted for at least 4 weeks. Although statistically significant, the increase in each function parameter was not as dramatic as from the previous study where miR-24 mimics were delivered virally (Fig. S3), suggesting additional beneficial effects might be executed through other cell types than cardiomyocytes.

### Reduced scar size in infarcted Myh6-miR-24 transgenic mice

**Fig. 4:**
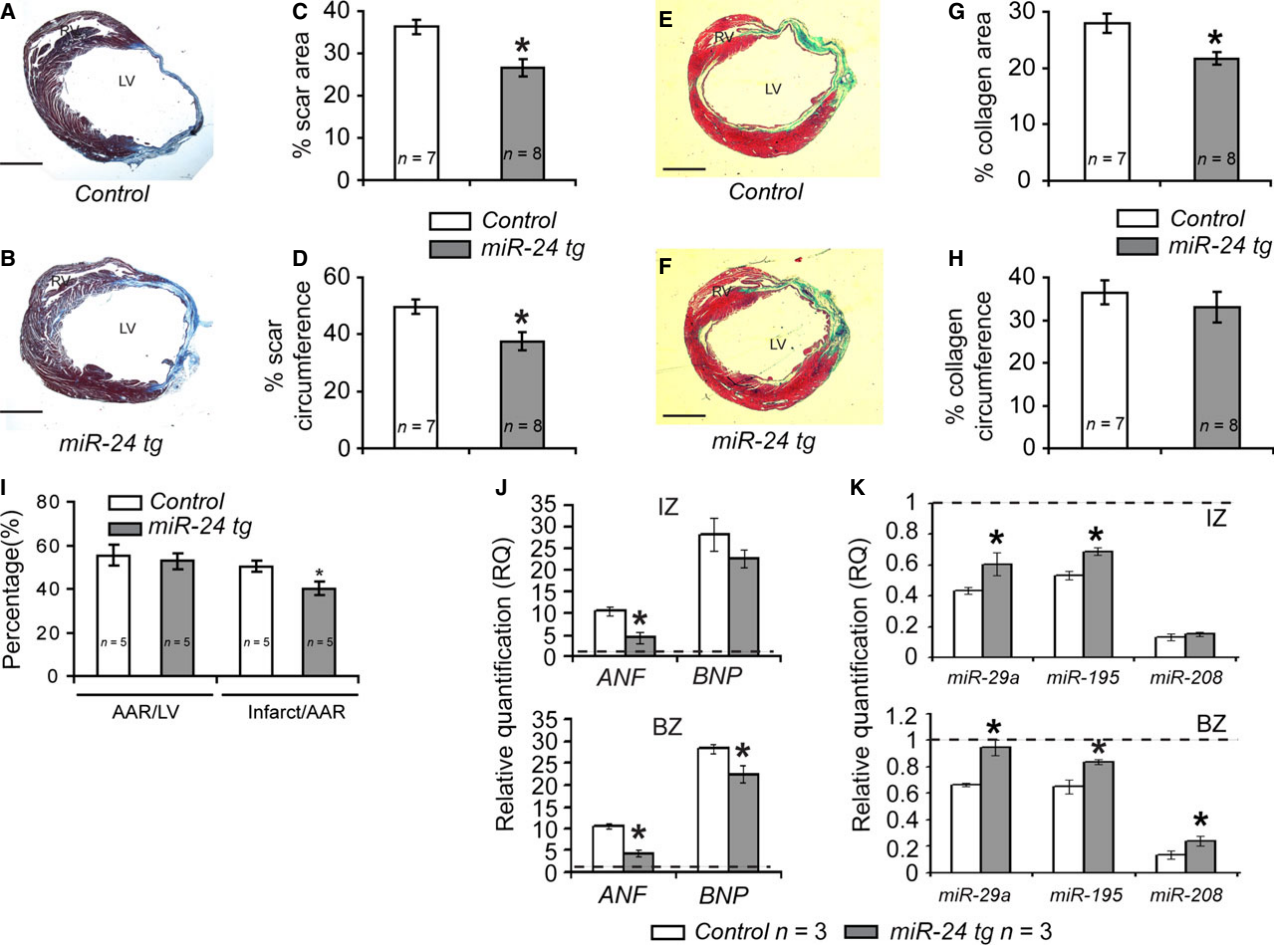
Myh6-miR-24 transgenic mice exhibit reduced scar size, lowered stress marker expression and partial restoration of dysregulated miRNAs. (**A**–**D**) Scar visualization by AZAN staining and scar size quantification on control (*n* = 7) and miR-24 tg (*n* = 8) hearts 4 weeks post-MI, with miR-24 tg mice showing reduced scar size. LV, left ventricle. (**E**–**H**) Alternative scar size visualization and quantification by Masson-Trichrome staining. Green = collagen; pink = cytoplasm; red = muscle fibres; black = nuclei. (**I**) Histogram showing size of area at risk (AAR) and size of infarct in control (*n* = 5) and miR-24 tg (*n* = 5) hearts 24 hrs after MI. miR-24 tg mice showed reduced infarct size. (**J**) Histograms showing relative expression levels of stress markers atrial natriuretic factor (ANF) and brain natriuretic peptide (BNP). miR-24 mice showed lowered levels of stress markers. IZ, infarct zone; BZ, boarder zone. (**K**) Histograms showing relative expression levels of microRNAs miR-29a, miR-195 and miR-208. miR-24 mice showed partial restoration of these miRNAs. *P < 0.05.

Cardiac fibrosis and scar formation are commonly resulted from ischaemia and shortage of oxygen in MI hearts. On the basis of the observation that cardiomyocyte death was attenuated and cardiac function was improved in Myh6-miR-24 transgenic mice, we tested if these would be correlated with reduction in scar size. As an important control experiment, we first determined if the inhibition of apoptosis by miR-24 expression in cardiomyocytes affected the degree of myocardial damage. We performed Evans blue/TTC double staining to assess the area at risk (AAR) and the infarct size of myocardium 24 hrs after coronary ligation. Overexpression of miR-24 led to a decrease in infarct size but no change in AAR (Fig. 4I), suggesting that miR-24 expression reduced cardiac damage shortly after MI. Four weeks later, we examined the scar size by performing AZAN staining on sections from multiple layers of LVs to delineate both viable myocardium and scar area (Fig. 4A and B). After blinded quantification on six sections from each of the four layers spanning LVs from seven control and eight transgenic mice, we found a reduction in both scar area and circumference in transgenic mice compared to controls (Fig. 4C and D). To further confirm our results, we performed Masson-Trichrome staining, which is another commonly used method for determining scar size in infarcted heart by differentially staining collagen (green), cytoplasm (pink), muscle fibres (red) and the nuclei (black; Fig. 4E and F). Although we did not detect statistically significant difference between control and transgenic hearts using the circumference of collagen area as the readout (Fig. 4H), we found that the difference became significant when using the area of collagen as the readout (Fig. 4G). As the calculation of area is typically more reliable than the calculation of circumference, potentially because of the irregular shape of certain scars, we believe the Masson-Trichrome data support our conclusion from AZAN staining. Meanwhile, it is important to notice the attenuation of scar formation is moderate compared to other published data where miR-24 was delivered non-genetically [24].

At the molecular level, miR-24 transgenic mice exhibited a lower level of stress markers such as atrial natriuretic factor (ANF) and brain natriuretic peptide (BNP), with partially restored levels of dysregulated miRNAs. We performed qPCR to detecct the expression levels of ANF, BNP and several stress-responsive miRNAs (miR-29, miR-195 and miR-208) in Myh6-miR-24 transgenic and control hearts. Both ANF and BNP were up-regulated 24 hrs after MI, while the up-regulation of ANF and BNP was attenuated in miR-24 transgenic hearts (Fig. 4J), which is similar to miR-24 mimic-treated hearts [24] (Fig. S4). Similarly, miR-29, miR-195 and miR-208 were dramatically reduced upon MI, but were partially restored by overexpression of miR-24 in cardiomyocytes (Fig. 4K).

Taken all together, we provide evidence that miR-24 improves LV function after MI by autonomous inhibition of cardiac cell death. However, the overall beneficial role of miR-24 in infarcted hearts is mild, and in most cases expression of miR-24 solely in cardiomyocytes is not able to revert the damaged myocardium close to normal.

### miR-24 modulates intrinsic apoptosis by suppressing Bim in a cell autonomous manner

**Fig. 5:**
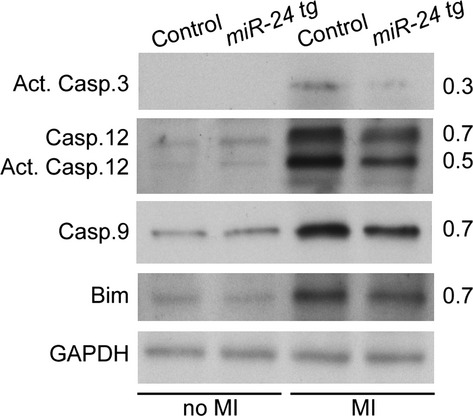
miR-24 regulates the intrinsic apoptosis pathways by suppressing the pro-apoptotic protein Bim. Western blot shows little expression of pro-apoptotic proteins Caspase 3, Caspase 12, Caspase 9 and Bim under normal physiological conditions (no MI) and elevated expression after myocardial infarction (MI). miR-24 mice showed reduced levels of pro-apoptotic proteins after MI compared to controls.

We have previously demonstrated that miR-24 inhibits cardiomyocytes apoptosis by targeting the pro-apoptotic Bcl2 family protein Bim [24]. Therefore, we tested if miR-24 regulated the same apoptosis pathway in our transgenic model where miR-24 is intrinsically overexpressed in cardiomyocytes. Proteins were extracted from the infarct and BZs of infarcted transgenic and wild-type mouse hearts, and then endogenous protein levels were analysed by Western blot. Activated Caspase 3 was barely detectable in both uninjured transgenic and littermate controls (Fig. 5 left). However, after MI there was a significant induction of Caspase 3 activity, which was less prominent in miR-24 transgenic hearts (Fig. 5 right). Similarly, without MI, no significant difference was observed for any caspase between transgenic mice and littermate controls (Fig. 5 left). However, the increases in the expression levels of the ER-specific caspase, Caspase 12 and the mitochondria-mediated apoptotic caspase, Caspase 9, were significantly attenuated in infarcted mouse hearts with miR-24 overexpression (Fig. 5 right). More importantly, the increase in protein level of Bim after MI was reduced in these hearts compared to controls (Fig. 5 right). Thus, we conclude that miR-24 inhibits Bim and regulates the intrinsic apoptosis in cardiomyocytes.

## Discussion

It has been demonstrated that miR-24 plays a role in a variety of cell types in the heart, including cardiomyocytes [24,36], fibroblasts [29], as well as endothelial cells [30]. This raises an interesting question about which cell type(s) is(are) the primary target of miR-24's protective functions in the heart, particularly under acute injury. Consistent with its expression in the adult cardiomyocyte, our study using a cardiac-specific transgenic model demonstrates that miR-24 could directly promote cardiomyocytes survival cell autonomously. We consistently observed that the mimic treatment has a stronger effect compared to transgenic approach. Interestingly, we found that although transgenic mice showed milder CM protection, the fold of overexpression is more consistent across areas in our genetic model. It seems that such transgenic approach offers a cleaner and more consistent way to promote the survival of CM upon injury albeit with a milder effect. Delivery of miRNAs using synthetic mimics, however, is more feasible for potential clinical application, while cautions have to be exercised considering the possible side effects of drastic miR-24 overexpression in the injection area. In addition, our data also suggest that miR-24 could have a cell non-autonomous effect when delivered into other cardiac cell types to promote the survival of the cardiomyocytes upon injury. Therefore, it will be interesting to test the pro-survival role of miR-24 in other cell types to device potential microRNA-therapy in additional tissues or organs.

microRNAs, given their unique features, typically regulate multiple targets in a very context-dependent manner. Like other microRNAs, miR-24 regulates a wide range of biological processes, and could exert seemingly opposite effects on different cell types. For example, miR-24 promotes apoptosis in human umbilical vein endothelial cells [30] and certain human cancer cell lines [37], while it inhibits apoptosis in other human cancer cell lines [38,39], rodent cardiomyocytes (mouse [24] and rat [40]), frog retinal cells [41] and mouse haematopoietic cells [42]. It is also interesting to note that miR-24 targets different apoptotic pathway components when positively or negatively regulating cell death. For example, pro-apoptotic genes such as *FAF-1* [39], *Caspase9* [24,41], *Bim* [24] and *Apaf-1* [41] were reported to mediate miR-24's anti-apoptotic function, while *PAK4* and *BAD* [30] were the targets that mediate miR-24's pro-apoptotic role. To elucidate miR-24's regulatory roles, it is important to ascertain its functions under different cellular environmental conditions. Identification of the context-dependent downstream targets is also necessary to fully understand miR-24's cell type-specific roles. In addition, characterization of co-regulators that restrict miR-24 to specific sets of targets in various tissues and cell types will undoubtedly help define the regulatory network of miR-24.

Both overexpression and inhibition of miR-24 have been reported to protect heart function upon injury [24,29], [30,31]. It is possible that the contradictory results may be because of the different types of viruses or synthetic oligos delivered into the hearts that may restrict miR-24 expression primarily in one cell type than the other. The time and location of delivery in the heart may also contribute to the discrepancy. To determine the role of miR-24 in the heart more precisely, we turned to genetic approach to specifically overexpress miR-24 in the cardiomyocytes, and demonstrated that miR-24 could directly prevent cardiomyocytes from undergoing apoptosis. It will be interesting to determine if a similar or opposite effect can be observed in endothelial specific miR-24 transgenics (*e.g*. Tie1-miR-24 or VE-cadherin-miR-24) and/or fibroblast specific miR-24 transgenics (*e.g*. Fsp1-miR-24 or Periostin-miR-24 or Tcf21-miR-24). In summary, characterizing how miR-24 promotes cell survival in the heart is a necessary next step for designing therapeutic strategies for cardiovascular diseases.

It will also be interesting to consider the difference in the timing of functional improvement between miR-24-mediated approach and reprogramming factors-mediated approach. It is observed that little or no improvement in cardiac function occurs until 8 weeks post-MI when reprogramming approaches are used to regenerate the heart [3], which is possibly attributable to the relatively long process of conversion. However, the prevention of cell death in cardiomyocytes resulted in a more immediate effect in functional improvement as early as 3 days after injury [24]. This information is particularly helpful when considering the suitability of each approach for different types of cardiac disease.

## Supporting information

**Figure S1** Myh6-miR-24 transgenic mice exhibit a more uniform miR-24 expression pattern compared to miR-24 mimic-treated mice. **Figure S2** Myh6-miR-24 transgenic mice exhibit heart function impairment after MI, but show protection compared to control littermates. **Figure S3** Myh6-miR-24 transgenic mice exhibit a milder protection effect compared to miR-24 mimic-treated mice after MI. **Figure S4** Gene expression of cardiac stress marker atrial natriuretic factor.
