# Supplementary figures and images for: Cardiomyocyte-specific role of miR-24 in promoting cell survival
Source: J Cell Mol Med. 2014 Oct 29;19(1):103–12. doi: 10.1111/jcmm.12393 (PMC4288354; doi:10.1111/jcmm.12393)

**Supplementary Figure 1**

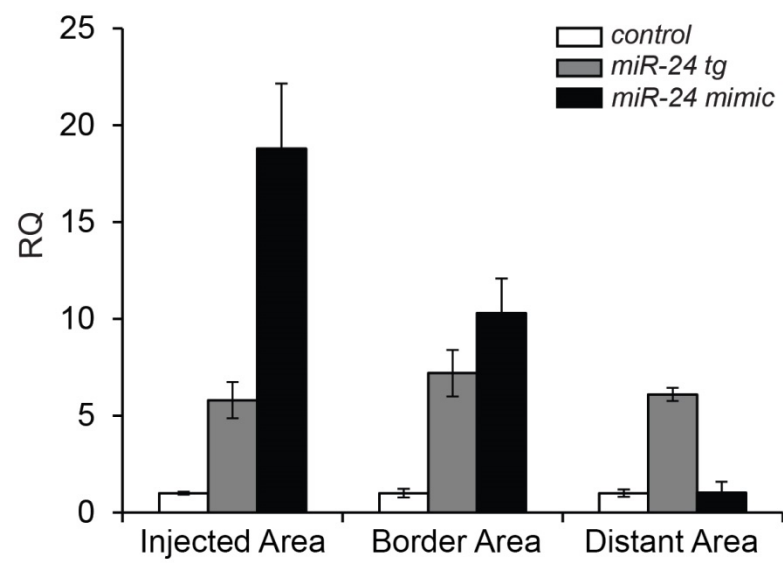

Supplementary Figure 2

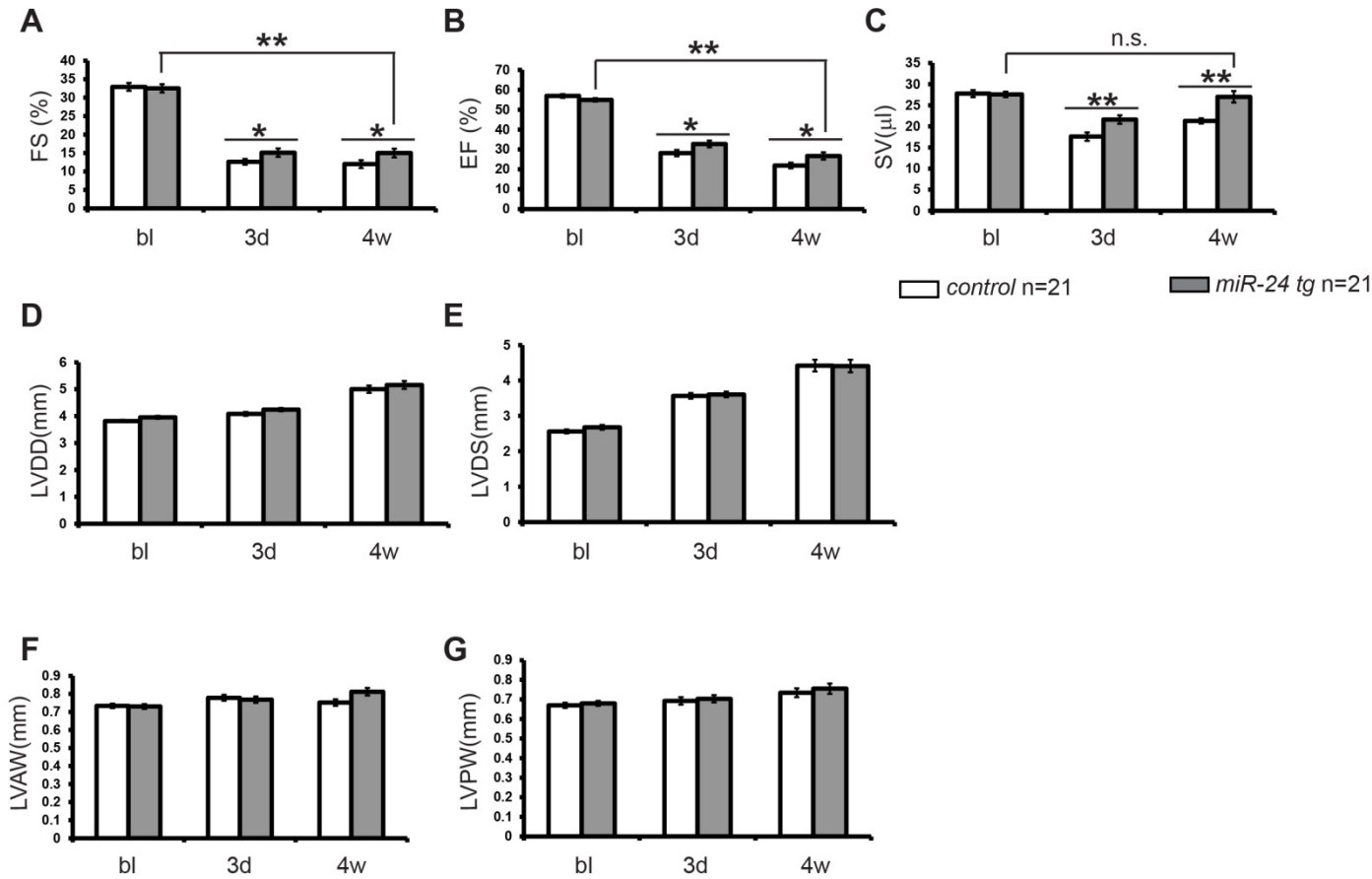

Supplementary Figure 3

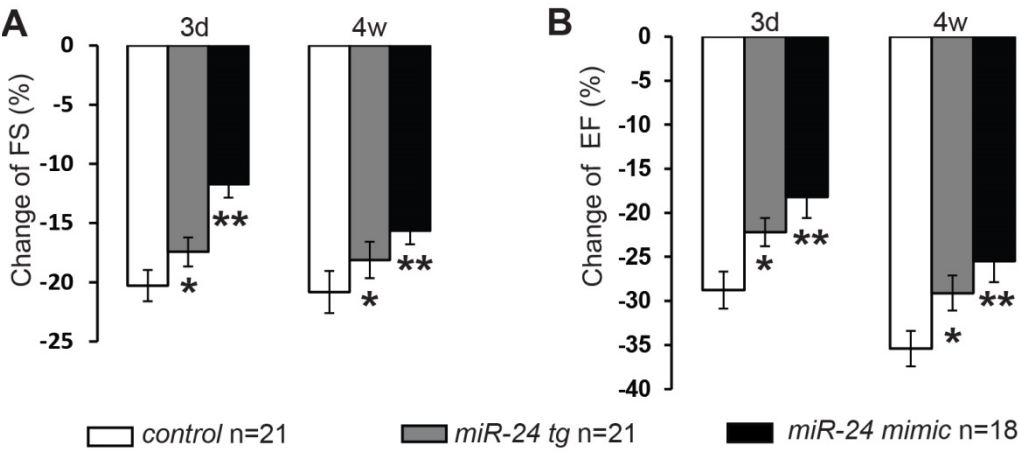

Supplementary Figure 4

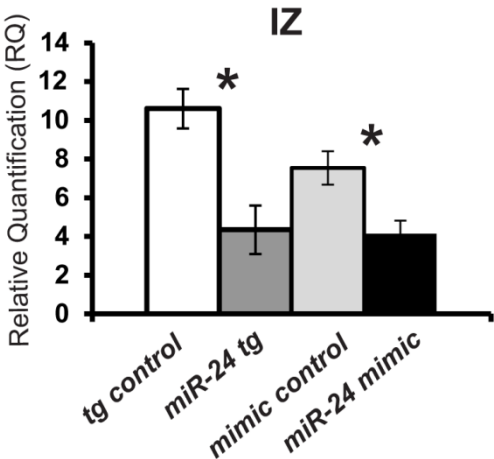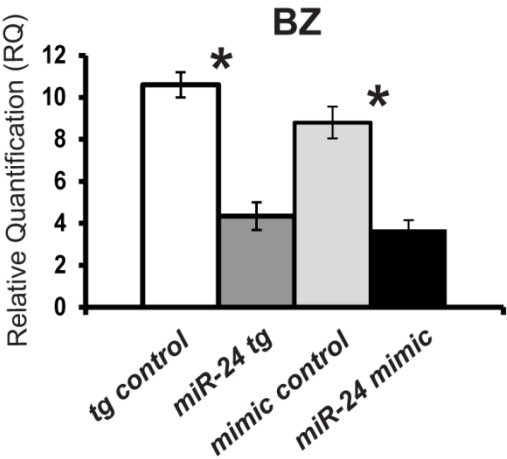

Supplement: Supplementary file 1 [file jcmm0019-0103-sd1.pdf]
